# Association of behaviour change techniques with effectiveness of dietary interventions among adults of retirement age: a systematic review and meta-analysis of randomised controlled trials

**DOI:** 10.1186/s12916-014-0177-3

**Published:** 2014-10-07

**Authors:** Jose Lara, Elizabeth H Evans, Nicola O’Brien, Paula J Moynihan, Thomas D Meyer, Ashley J Adamson, Linda Errington, Falko F Sniehotta, Martin White, John C Mathers

**Affiliations:** Human Nutrition Research Centre, Newcastle University, Biomedical Research Building, Campus for Ageing and Vitality, Newcastle upon Tyne, NE4 5PL UK; Institute of Cellular Medicine, Newcastle University, Newcastle, UK; Institute of Health and Society, Newcastle University, Newcastle, UK; Department of Psychiatry & Behavioral Sciences, University of Texas Health Science Center, Houston, TX USA; Centre for Oral Health Research, Newcastle University, Newcastle, UK; Fuse, UKCRC Centre for Translational Research in Public Health, Newcastle, UK; Walton Library, Newcastle University, Newcastle, UK; Centre for Brain Ageing and Vitality, Newcastle University, Newcastle, UK

**Keywords:** Behaviour change techniques, Fruit and vegetables, Retirement, Aging, Randomised controlled trial, Systematic review, Meta-analysis

## Abstract

**Background:**

There is a need for development of more effective interventions to achieve healthy eating, enhance healthy ageing, and to reduce the risk of age-related diseases. The aim of this study was to identify the behaviour change techniques (BCTs) used in complex dietary behaviour change interventions and to explore the association between BCTs utilised and intervention effectiveness.

**Methods:**

We undertook a secondary analysis of data from a previous systematic review with meta-analysis of the effectiveness of dietary interventions among people of retirement age. BCTs were identified using the reliable CALO-RE taxonomy in studies reporting fruit and vegetable (F and V) consumption as outcomes. The mean difference in F and V intake between active and control arms was compared between studies in which the BCTs were identified versus those not using the BCTs. Random-effects meta-regression models were used to assess the association of interventions BCTs with F and V intakes.

**Results:**

Twenty-eight of the 40 BCTs listed in the CALO-RE taxonomy were identified in the 22 papers reviewed. Studies using the techniques ‘barrier identification/problem solving’ (93 g, 95% confidence interval (CI) 48 to 137 greater F and V intake), ‘plan social support/social change’ (78 g, 95%CI 24 to 132 greater F and V intake), ‘goal setting (outcome)’ (55 g 95%CI 7 to 103 greater F and V intake), ‘use of follow-up prompts’ (66 g, 95%CI 10 to 123 greater F and V intake) and ‘provide feedback on performance’ (39 g, 95%CI −2 to 81 greater F and V intake) were associated with greater effects of interventions on F and V consumption compared with studies not using these BCTs. The number of BCTs per study ranged from 2 to 16 (median = 6). Meta-regression showed that one additional BCT led to 8.3 g (95%CI 0.006 to 16.6 g) increase in F and V intake.

**Conclusions:**

Overall, this study has identified BCTs associated with effectiveness suggesting that these might be active ingredients of dietary interventions which will be effective in increasing F and V intake in older adults. For interventions targeting those in the peri-retirement age group, ‘barrier identification/problem solving’ and ‘plan for social support/social change’ may be particularly useful in increasing the effectiveness of dietary interventions.

**Electronic supplementary material:**

The online version of this article (doi:10.1186/s12916-014-0177-3) contains supplementary material, which is available to authorized users.

## Background

Adopting healthier dietary patterns is central to the prevention of non-communicable chronic diseases and promotion of healthy ageing [1-3]. The worldwide mortality attributable to low consumption of fruits and vegetables (F and V) is estimated to be 2.635 million deaths per year and increasing per capita F and V consumption to 600 grams per day could reduce the risk of ischaemic heart disease and ischaemic stroke, and of several types of cancer [4] and so reduce total worldwide burden of disease by 1.8%. Thus, the development and implementation of effective dietary interventions have considerable potential to improve health in later life but interventions specifically targeting people of retirement age are lacking. In a recent critical analysis of the evidence on the effectiveness of dietary interventions in those 54- to 70-years old we reported that dietary interventions in this life stage produce increases in F and V intake of 88 g/day which are sustainable in the longer term and likely to be of public health significance [5]. However, there was considerable heterogeneity only partially associated with type of intervention, study design, ethnicity, sex, geographic origin of studies and mode of delivery of interventions. These findings together with previous analysis of interventions delivered in primary care settings [6,7], suggests that there is a need for better understanding of the effective components of such behavioural interventions. Potentially important intervention features include the number of contacts with participants, which are associated with greater, more sustained increases in F and V intake and other dietary and lifestyle improvements [5-7]. In addition, the use of particular behaviour change theories and techniques has been previously highlighted as critical aspects of interventions development [8]. Identification of the most effective behaviour change techniques (BCTs) associated with improved outcomes in lifestyle interventions [9] would have considerable utility in informing the development of effective future interventions.

The development of standardised definitions of BCTs, notably the CALO-RE taxonomy, a standardised 40-item taxonomy which can be used to identify BCTs reliably in reports of intervention studies [10], has provided a useful framework for understanding how intervention content is associated with intervention effectiveness. This taxonomy has been applied in recent systematic reviews aiming to identify effective BCTs in increasing physical activity in obese individuals [11] and older subjects [12], limiting gestational weight gain [13,14], promoting weight loss in adults [15] and preventing and managing childhood obesity [16]. However, this approach has not yet been applied in analyses of the effectiveness of dietary interventions. Recently, we systematically reviewed and meta-analysed the evidence on dietary interventions promoting the Mediterranean diet (MD) or any of its components (for example, fruits and vegetables (F and V)), among adults of retirement transition age, with at least three months follow up [5]. Retirement from work is one of the last transitional life events involving important lifestyle changes and may represent a window of opportunity to promote healthier eating patterns in later life. However, the active ingredients associated with greater effectiveness of dietary interventions among this population group remain to be identified. Here, we present a further analysis of those studies in which we aimed to identify the BCTs that are associated with more effective dietary interventions with a particular focus on F and V intake. We also evaluated whether behaviour theories were explicitly mentioned in these studies and their association with intervention effectiveness.

## Methods

In a recent systematic review of dietary interventions focusing on the MD or any of its food components and targeting people of retirement age (defined as a study mean or median age between 54 to 70 years), we identified F and V intake as the most commonly reported dietary outcome [5]. The protocol for the original systematic review was registered with PROSPERO, the International Prospective Register of Systematic Reviews (Registration number CRD42011001484). Our systematic review is reported according to PRISMA [17] and the detailed methodology together with the PRISMA flow chart and checklist of the original review have been described elsewhere [5], so only pertinent details are reported here.

In March 2013, 12 electronic databases, namely Medline, Embase, PsycInfo, Scopus, Web of Science, CINAHL, ASSIA, Cochrane Database of Systematic Reviews, CAB Abstracts, Conference Papers Index, WorldCat Dissertations database and Index to Theses, were searched for randomised controlled trials (RCTs) of interventions promoting healthy dietary patterns, such as the MD or any of its component food groups (for example, F and V; legumes or pulses; nuts and seeds; unrefined cereals; olive oil; fish; moderate consumption of wine; low consumption of meat and meat products). To measure sustained behaviour change, only intervention studies with a follow-up of ≥3 months were included. The primary outcome was dietary change and this analysis focuses on the fruit and/or vegetable intake (in grams per day) since this was the outcome common to all studies reviewed. The literature searches yielded 22 studies which provided data for meta-analysis and for evaluating behaviour change features of these interventions [18-39].

### Behaviour change techniques

We used the CALO-RE taxonomy [10], a reliable 40-item taxonomy of standardised BCT definitions used in physical activity and healthy eating interventions, which has been used recently to evaluate potentially active ingredients in complex behaviour change of interventions [11,13,14,16]. Intervention content from each of the 22 articles was reviewed and coded for the presence (or absence) of each of the 40 BCTs in the CALO-RE taxonomy. When a protocol of the study was published, this was also evaluated. Two reviewers (JL and EE) were trained by an experienced health psychologist (NH) in the use of the BCT-taxonomy. The two reviewers (JL and EE) coded the intervention content independently and discrepancies between them were resolved through discussion with a third reviewer (NH) at each stage of the review process and a consensus approach used. Behaviour change theories used in these studies, if explicitly declared, were also recorded.

### Statistical analysis

Review Manager (RevMan Version 5.1 for Windows Copenhagen: The Nordic Cochrane Centre, The Cochrane Collaboration, 2011) and Stata (Stata/SE 11.2 for Windows; StataCorp LP, College Station, TX, USA) were used to pool and analyse results from the individual studies.

Pooled results are reported as mean differences with 95% confidence intervals (Cis) and with two-tailed *P*-values. A random effects model accounting for inter-study variation was used, thereby minimising potential bias due to methodological differences between studies. Multiple dietary intervention arms from three studies were included in the meta-analysis. As suggested by Higgins *et al*. [40] excessive weightings from ‘double counts’ originating from the ‘shared’ group (that is, control group), were controlled by splitting the sample size of the shared group into approximately equal smaller groups for the comparisons; the means and standard deviations were left unchanged. When available, we used results from multivariate models with the most complete adjustment for potential confounders reported in original studies. Analysis was performed to investigate associations between BCTs identified in complex interventions and in the effectiveness of the interventions in increasing F and V intake by comparing effectiveness of those studies which did, and those which did not, employ the specified BCT. To increase statistical power and reduce the likelihood of type-1 error associated with multiple comparisons we limited this analysis to the most frequently reported BCTs (that is, those BCTs which were reported in at least five studies).

Statistical heterogeneity was evaluated by using the *I*^2^ statistic [40,41]; as proposed by Ioannidis *et al.* [42] the 95% CIs for *I*^2^ were calculated using the Higgins *et al*. method [43]. Where *I*^2^ was >50%, the degree of heterogeneity was considered high.

Publication bias was appraised by visual inspection of a funnel plot of effect size against the standard error (SE) for each study, with asymmetry assessed formally with Egger’s regression test [44].

We performed meta-regression analysis with restricted maximum likelihood estimation to assess the relationship of the number of behaviour techniques extracted from each study with changes in F and V consumption.

## Results

Twenty-two studies provided data for meta-analysis and coding of BCTs [18-39]. The pooled study populations included 63,189 participants who were followed-up for 19 months on average (range 4 to 58 months). The mean ages of participants in these studies ranged from 54 to 67 years. Four studies recruited women only and two men only (see Additional file 1). A funnel plot of the mean differences against SEs of all studies did not indicate important asymmetry, which was confirmed by the Egger’s regression test (*P* = 0.394).

### Behaviour change techniques meta-analysis

Twenty-eight out of 40 BCTs in the CALO-RE taxonomy were identified in the studies reviewed. The five most commonly reported BCTs were ‘goal setting (behaviour)’, ‘provide information on consequences of behaviour *in general*’, ‘provide instruction on how to perform the behaviour’, ‘provide feedback on performance’ and ‘goal setting (outcome)’ (Table 1). The 12 BCTs from the CALO-RE taxonomy which were not identified in the reviewed studies were ‘provide information about others’ approval’, ‘prompt review of outcome goals’, ‘prompt rewards contingent on effort or progress towards behaviour’, ‘shaping’, ‘prompting generalisation of a target behaviour’, ‘prompting focus on past success’, ‘prompt identification as role model/position advocate’, ‘prompt anticipated regret’, ‘fear arousal’, ‘prompt use of imagery’, ‘general communication skills training’ and ‘stimulate anticipation of future rewards’. The number of incorporated BCTs per study ranged from 2 to 16, with a median of 6 BCTs per study.

**Table 1:** **Frequency of use of the behaviour change techniques in the 22 studies reviewed**

**(CALO-RE taxonomy item) Behaviour change techniques identified**	**Number of studies or subgroups**
(5) Goal setting behaviour	23
(1) Provide information on consequences of behaviour in *general*	17
(21) Provide instruction on how to perform the behaviour	14
(19) Provide feedback on performance	13
(6) Goal setting outcome	10
(2) Provide information on consequences of behaviour *to the individual*	9
(27) Use of follow-up prompts	8
(8) Barrier identification/problem solving	7
(37) Motivational interviewing	7
(22) Model/Demonstrate the behaviour	6
(29) Plan social support/social change	6
(10) Prompt review of behavioural goals	5
(24) Environmental restructuring	4
(7) Action planning	3
(28) Facilitate social comparison	3
(16) Prompt self-monitoring of behaviour	3
(23) Teach to use prompts/cues	3
(26) Prompt practice	2
(4) Provide normative information about others’ behaviour	2
(13) Provide rewards contingent on successful behaviour	2
(25) Agree behavioural contract	1
(17) Prompt self-monitoring of behavioural outcome	1
(20) Provide information on *where and when* to perform the behaviour	1
(35) Relapse prevention/coping planning	1
(33) Prompt self talk	1
(9) Set graded tasks	1
(36) Stress management/emotional control training	1
(38) Time management	1

**Figure 1:**
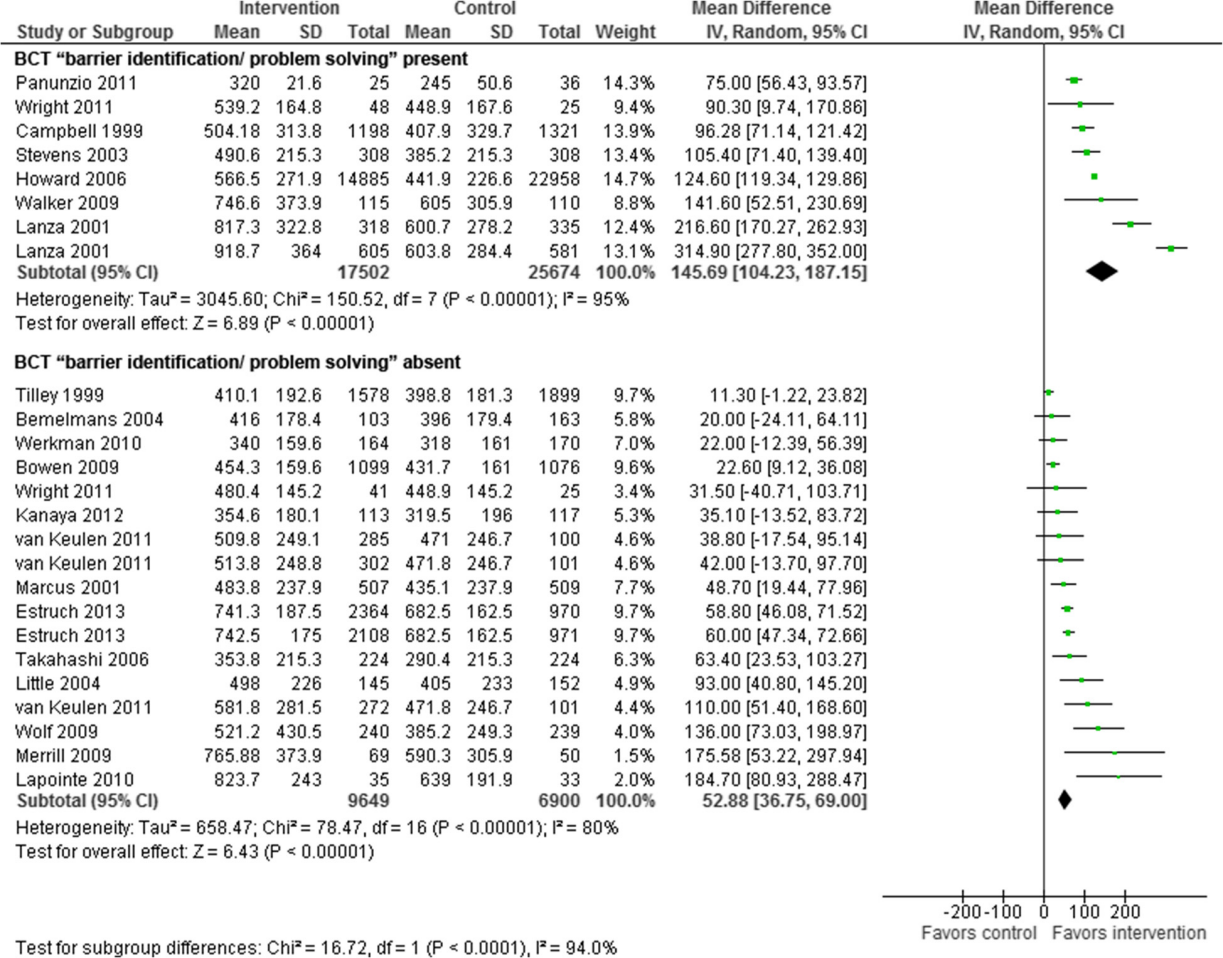
**F and V intakes in 22 RCTs by the presence or absence of the ‘barrier identification/problem solving’ BCT.** Mean differences and 95%CI are for fruit and vegetable (F and V) intakes in grams/day. Mean difference of Mean differences in F and V (95%CI) = 93 (48 to 137). BCT, behaviour change technique; CI, confidence interval; RCT, randomized controlled trial.

**Figure 2:**
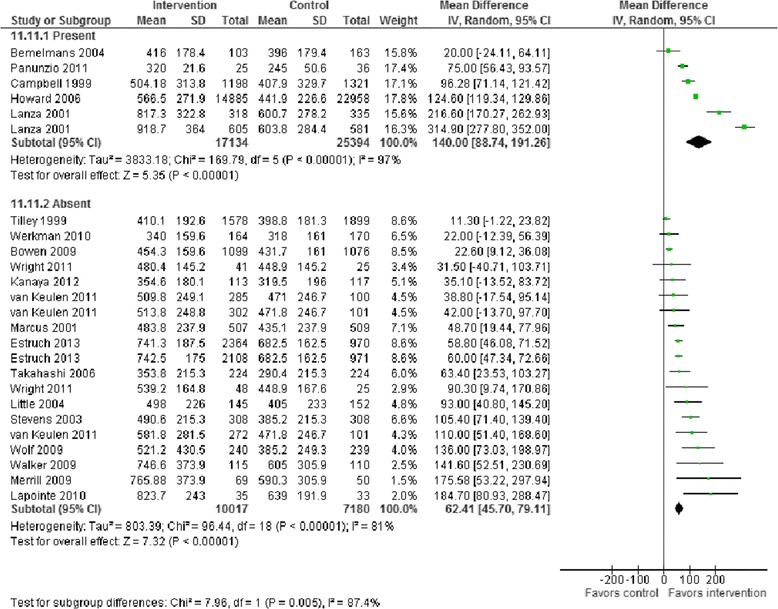
**F and V intakes in 22 RCTs by the presence or absence of the ‘plan for social support/social change’ BCT.** Mean differences and 95%CI are for fruit and vegetable intakes in grams/day. Mean difference of Mean differences in F and V (95%CI) = 78 (24 to 132). BCT, behaviour change technique; CI, confidence interval; RCT, randomized controlled trial.

Meta-analysis of change in intake of F and V in response to intervention was carried out based on the presence or absence of specific BCTs which were reported in at least five RCTs. Results showed that studies incorporating the BCTs ‘barrier identification/problem solving’ (93 g, 95%CI 48 to 137 greater F and V intake) (Figure 1), ‘plan social support/social change’ (78 g, 95%CI 24 to 132 greater F and V intake) (Figure 2), ‘use of follow-up prompts’ (66 g, 95%CI 10 to 123 greater F and V intake) and ‘goal setting (outcome)’ (55 g 95%CI 7 to 103 greater F and V intake) (Table 2) were associated with conclusive and clinically important improvements in F and V intakes. In addition, there was weaker evidence that studies employing the BCT ‘provide feedback on performance’ were associated with greater F and V intake than studies not incorporating this BCT in their interventions (between groups difference 39 g 95%CI −2 to 81) (Table 2). Conversely, studies reporting use of the BCTs ‘motivational interviewing’ and ‘provide information on consequences of behaviour to the individual’ were associated with significantly smaller improvements in F and V intakes in comparison with studies not using these BCTs (Table 2). Forest plots for each of the BCTs investigated in Table 2 are provided as supplementary material [see Additional file 1].

**Table 2:** **Association of behaviour change techniques (BCTs) with fruit and vegetable intakes in 22 intervention studies**

**BCT**	**Number of studies or subgroups BCT present Yes (No)**	**Sample size BCT present Yes (No)**	**BCT present Mean difference (95% ** **CI)**	**I** ^**2**^ **(95% ** **CI)**	**BCT absent Mean difference (95%** **CI)**	**I** ^**2**^ **(95% ** **CI)**	**(Mean difference of Mean differences (95%CI))** ***P***
**Motivational interviewing**	7 (18)	10,183 (49503)	52.8 (33.1 to 72.8)	81 (61 to 90)	105.1 (71.4 to 138.7)	97 (95 to 97)	(−52 (−91 to −13)) 0.009
**Provide information on consequences of behaviour to the individual**	11 (14)	12,488 (9394)	60.1 (45.0 to 75.3)	62 (27 to 80)	109.6 (64.6 to 154.9)	96 (95 to 97)	(−49 (−97 to −2)) 0.040
**Prompt review of behavioural goals**	5 (20)	7,163 (52562)	69.1 (55.8 to 82.3)	53 (0 to 83)	92.9 (59.7 to 126.1)	97 (96 to 98)	(−24 (−60 to 12)) 0.190
**Goal setting (behaviour)**	23 (2)	59,323 (402)	89.0 (63.6 to 114.5)	97 (96 to 98)	95.7 (−63.1 to 254.4)	88 (N/A)	(−7 (−166 to 153)) 0.940
**Model/Demonstrate the behaviour**	6 (19)	3,258 (56467)	91.3 (56.9 to 125.7)	71 (32 to 87)	85.0 (55.9 to 114.2)	95 (97 to 98)	(6 (−39 to 51)) 0.790
**Provide instruction on how to perform the behaviour**	15 (10)	53,717 (6008)	99.5 (69.6 to 129.5)	97 (96 to 98)	67.8 (34.5 to 101.2)	78 (59 to 88)	(32 (−13 to 77)) 0.170
**Provide information on consequences of behaviour in general**	18 (7)	53,298 (6427)	97.8 (67.6 to 127.9)	97 (96 to 98)	66.7 (32.3 to 101.1)	86 (74 to 93)	(31 (−15 to 77)) 0.180
**Provide feedback on performance**	14 (11)	54,563 (5162)	102.5 (68.9 to 136.0)	98 (97 to 98)	63 (37.9 to 88.0)	78 (60 to 87)	(39 (−2 to 81)) 0.060
**Goal setting (outcome)**	11 (14)	43,702 (16023)	118.1 (74.3 to 161.9)	97 (96 to 98)	62.8 (43.5 to 82.2)	83 (73 to 90)	(55 (7 to 103)) 0.020
**Use of follow-up prompts**	9 (16)	41,627 (18098)	127.4 (74.1 to 180..7)	95 (92 to 97)	60.9 (43.4 to 78.4)	86 (78 to 91)	(66 (10 to 123)) 0.020

Mean differences and 95%CI are for fruit and vegetable (F and V) intakes in grams/day. *P*-values correspond to Chi square test for difference between groups (that is, BCT present versus BCT absent). CI, confidence interval.

**Figure 3:**
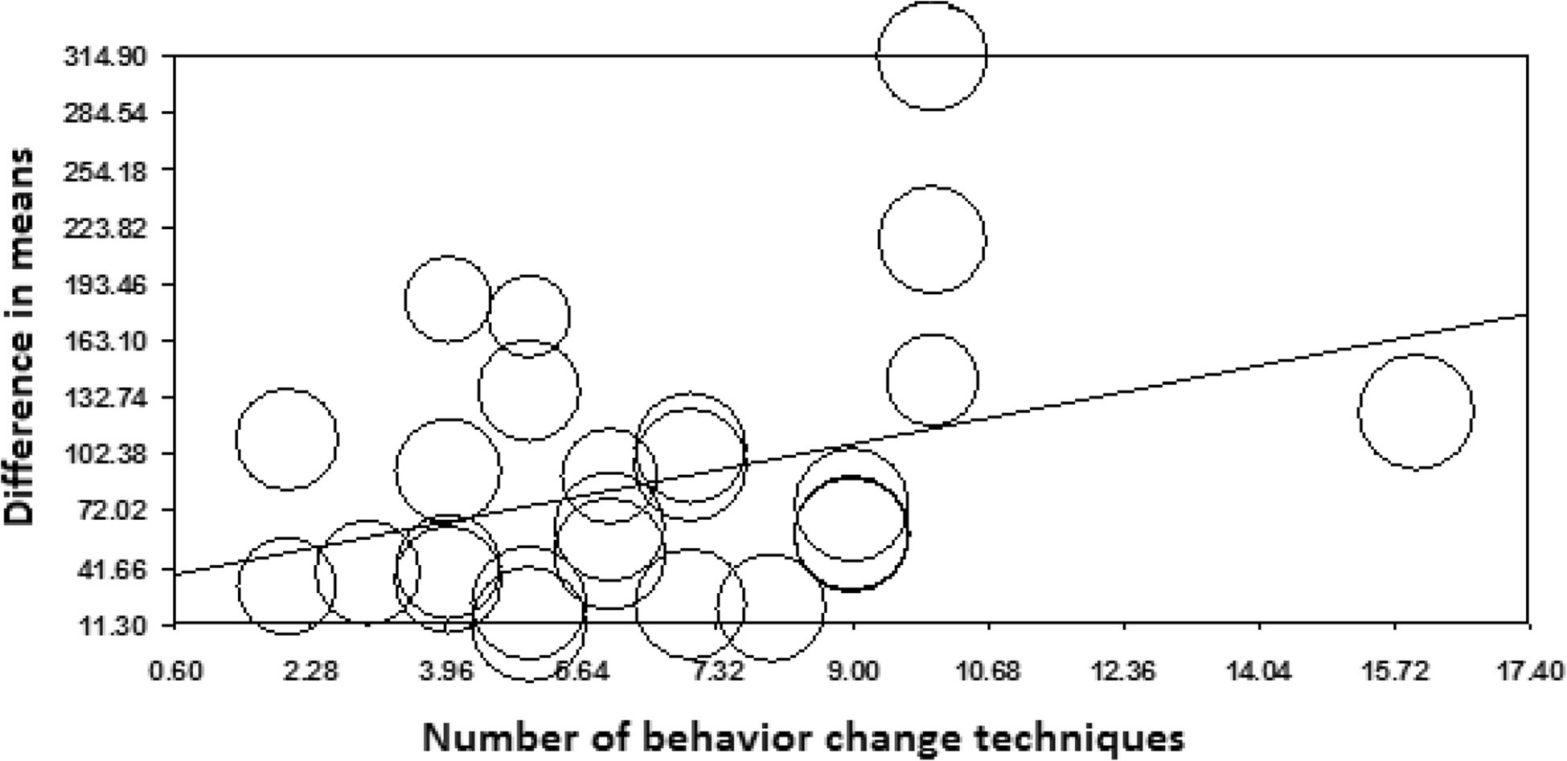
**Meta-regression of number of BCTs on overall fruit and vegetable intake.** Number of BCTs. Slope = 8.28, Q = 3.84, d.f. = 1, *P* = 0.049. The circle size reflects the weight that a study obtained in the meta-regression.

Meta-regression showed that one additional BCT led to 8.3 g (95%CI 0.006 to 16.6 g) increase in F and V intake (Figure 3).

### Theoretical basis of interventions

Fourteen studies reported the use of behaviour change theories in the development of their intervention and eight studies did not report use of any theory. Eight of the 14 studies reported use of a combination of two or more behaviour change theories while the other six studies reported using a single theory. The behaviour change theories declared in the reviewed studies included social cognitive theory (n = 5), social learning theory (n = 4), the transtheoretical model (n = 4), theory of planned behaviour (n = 2), social support (n = 2), and health behaviour model (n = 2), self-management (n = 1). Subgroup analysis showed that studies (n = 14) reporting the use of behaviour change theories reported changes in F and V intake which were nearly 50% greater (94.7 g/day 95%CI 49.1 to 140.3 g) than in those studies not reporting use of a behaviour change theory (66.5 g/day 95%CI −7.7 to 140.7 g) but the difference between these groups (28.2 g 95%CI −58.9 to 115.3) was not statistically significant. Studies reporting the use of a single behaviour change theory achieved 67% greater increase mean F and V intakes (n = 6) (121.6 g/day 95%CI 56.8 to 186.5 g) than those studies reporting use of a combination of behaviour change theories (n = 8) (72.8 g/day 95%CI 11.9 to 133.7) but the difference between these groups (48.8 g 95%CI −40.1 to 137.7) was not statistically significant.

## Discussion

### Statement of principal findings

This analysis suggests that use of BCTs, including barrier identification/problem solving, plan social support/social change, use of follow-up prompts and goal setting (outcome), is associated with enhanced effectiveness of dietary interventions. Using these BCTs in the intervention design resulted in increases in F and V intakes which were 60 to 90 g/day greater than when these BCTs were not utilised in the intervention.

### Strengths and weaknesses of the study

To our knowledge, this is the first study evaluating the association of BCTs use in dietary interventions in promoting sustained increases in F and V intake among individuals of retirement transition age. The strengths of this study include a rigorous methodology in the systematic review of the literature and the application of a well-established BCT taxonomy to identify BCT use. Although it is possible that the findings of this study might be confounded by unexplored covariates, previous analysis published elsewhere [5] showed that other aspects of the populations or interventions reviewed, such as mode of delivery of the intervention, health status of participants, follow-up time or ethnicity, were not related to F and V intakes and, therefore, are unlikely to confound the present findings. In addition, we limited the effect of multiple comparisons by restricting the comparisons to only those BCTs observed in five or more studies. Among the limitations are the small number of studies (n = 22) available and the high level of unexplained heterogeneity in this meta-analysis. In addition, from the studies reviewed we cannot confirm that the BCTs identified in these interventions were delivered as intended. The subgroup analyses are exploratory and the results obtained should be interpreted with caution because of the small numbers of studies in each sub-group and the *post hoc* nature of the analysis. The limited number of studies available determined that our analysis focused on examining the associations between F and V intake in studies with the presence or absence of specific BCTs and associations of combinations of BCTs with intervention effects on F and V intake could not be evaluated.

### Scientific analysis of findings

Research into the effectiveness of including specific BCTs in dietary interventions is still limited. Using an earlier version of the BCT taxonomy comprising 26 BCTs, Michie *et al*. [9], reported that the inclusion of techniques derived from Control Theory (‘prompt intention formation’, ‘prompt specific goal setting’, ‘prompt review of behavioural goals’, ‘prompt self-monitoring of behaviour’, ‘provide feedback on performance’) was associated with increased effectiveness of interventions of healthy eating and physical activity compared with those which did not include such techniques.

To our knowledge, no other systematic review has evaluated the use of the CALO-RE taxonomy on the effectiveness of dietary interventions. However, four published systematic reviews, focusing on physical activity [11,12], gestational weight gain [13,14], prevention and management of childhood obesity [16] and weight management [15] have used the CALO-RE taxonomy. Gardner *et al*. [13] found no obvious differences in the BCTs employed between effective and ineffective interventions, while French *et al*. [12] reported that self-regulatory techniques were associated with lower levels of both self-efficacy and physical activity. The remaining reviews have identified a range of BCTs but only ‘prompt practice’ [11,16] and ‘provision of information on the consequences of behaviour to the individual’ [14,16] have been identified repeatedly in two systematic reviews. In addition, only Hartmann-Boyce *et al*. [15], reported BCTs associated with lower effectiveness of interventions; these were ‘prompting focus on past success’ and ‘prompt self-talk’ (Table 3).

**Table 3:** **Published systematic reviews applying the CALO-RE taxonomy of behaviour change techniques**

**Outcome reviewed**	**Reference**	**BCTs identified as with greater effectiveness of interventions**	**BCTs identified as with lower effectiveness of interventions**
Increasing physical activity in obese individuals	Olander (2013) [11]	• ‘teach to use prompts/cues’	
		• ‘prompt practice’	
		• ‘prompt rewards contingent on effort or progress towards behaviour’	
	French (2014) [12]	• None	• ‘setting behavioural goals’
			• ‘prompting self-monitoring of behaviour’
			• ‘planning for relapses’
			• ‘providing normative information’
			• ‘providing feedback on performance’
Limiting gestational weight gain	Gardner (2011) [13]	• No obvious differences in the behaviour change techniques employed between effective and ineffective interventions	
	Hill (2013) [14]	• ‘provision of information on the consequences of behaviour to the individual’	
		• ‘motivational interviewing’	
		• ‘behavioural self-monitoring’	
		• ‘providing rewards contingent on successful behaviour’	
Promoting weight loss in adults	Hatmann-Boyce (2014) [15]	• ‘provide information about others’ approval’	• ‘prompting focus on past success’
		• ‘provide normative information about others behaviour’	• ‘prompt self-talk’
		• ’model/demonstrate the behaviour’	
		• ‘facilitate social comparison’	
Preventing and managing childhood obesity	Martin (2013) [16]	• ‘provide information on the consequences of behaviour to the individual’	
		• ‘environmental restructuring’	
		• ‘prompt practice’	
		• ‘prompt identification as role model/position advocate’	
		• ‘stress management/emotional control training’	
		• ‘general communication skills training’	

None of these systematic reviews, including ours, replicated the findings of a clear clustering of BCTs associated with the Control Theory as the key BCTs associated with better dietary outcomes reported by Michie *et al*. [9]. Of the five BCTs identified in the present study, only ‘plan social support/social change’ was also identified by Olander *et al*. [11] as associated with positive changes in physical activity self-efficacy among obese individuals. The median number of BCTs, as well as the most frequently reported BCTs, identified in the present study are similar to the BCTs identified in these studies [11,13,14,16]. Differences in the effective BCTs identified in different reports might result from differences in the population groups studied; therefore, specific BCTs useful in a particular population group might not be equally useful in a different population group.

BCTs congruent with the Control Theory were associated with greater effectiveness of lifestyle interventions in the report by Michie *et al*. [9] but the present study revealed four BCTs which were effective in increasing F and V and which may also be part of several behaviour change theories. The two BCTs which were more strongly associated with improved F and V intakes in our systematic review, ‘barrier identification/problem solving’ and ‘social support’ are congruent with the Social Cognitive Theory [45]. In addition, significant, but more moderate, effects on intervention effectiveness were observed for BCTs congruent with the Operant Learning Theory [46]. However, it is important to note that individual BCTs are not exclusive to specific behaviour change theory.

Analysis of current evidence on the impact of the extent and type of theory on diet and physical activity interventions suggests that theory is not often used or reported and that its relationship with intervention effectiveness is weak [47]. Hill *et al*. [14] found that studies based on theory were as effective as non–theory-based studies at limiting gestational weight gain. These findings are supported by our observation of no significant differences in effectiveness of theory-based versus non theory-based interventions.

Our results showed a significant trend towards greater improvements in F and V intake with employment of more BCTs per study. This finding, however, contradicts previous evidence suggesting that it might be more important to focus on a few effective BCTs, rather than a greater number of BCTs, when designing dietary interventions [9,13,14].

Twelve BCTs from the CALO-RE taxonomy were not identified in any of the dietary interventions reviewed here, while 16 BCTs were reported by fewer than 5 studies and, therefore, were not considered in this analysis. More research on the potential usefulness of this sub-set of less frequently used BCTs in improving the effectiveness of dietary interventions is required.

Previously, we reported a significant positive association between number of contacts within the intervention and greater increases in F and V intakes, suggesting the importance of features of interventions on their effectiveness [5]. These results are supported by a recent report from the U.S. Preventive Services Task Force (USPSTF) on behavioural counselling to promote a healthful diet and physical activity in adults without pre-existing cardiovascular disease (CVD) or its risk factors suggesting that interventions of moderate to high-intensity, but not low-intensity, were associated with better results [6,7].

### Implications for health and policymaking

Worldwide increases in life expectancy are increasing the proportion of older adults in most populations with consequent increases in the proportion of retirees [48]. Retirement has been associated with increases in body weight and waist circumference, well known risk factors for CVD [49], and health promotion at retirement is of considerable importance to retirees and policy makers [50]. The findings of this study will contribute to the development of effective dietary interventions incorporating BCTs that have proven useful among adults of retirement age. Interventions which incorporated ‘barrier identification/problem solving’ and ‘plan social support/social change’ BCTs resulted in approximately 93 and 78 g/day extra F and V, respectively, (equivalent to about a portion of F and V according to UK standards [51]) compared with those not using these BCTs. Based on average population intakes of F and V in the UK (4.2 portions or 336 g/day, in adults 19 to 64 years; 4.4 portions or 352 g/day in older adults 65-years old and older), interventions incorporating more effective BCTs could make a significant difference in achieving the WHO recommendation of at least 400 g F and &V/day [52].

Several systematic reviews and meta-analyses of cohort studies indicate that small differences in F and V intake, such as the ones observed in the present study, are associated with reductions in the risk for cardiovascular events with significant potential for population impact. Increasing F and V consumption by around 100 to 150 g/day decreases the risk of stroke by 11% [53,54], coronary heart disease by 4% to 7% [54,55] and diabetes by 10% to 14% [56,57]. Future research should test the effect of tailored interventions using the BCTs identified in this systematic review. Our study focussed on those 54- to 70-years old, and whether the BCTs identified in this study are applicable to other age groups remains to be tested. The CALO-RE taxonomy of BCTs [10] is a useful tool and its adoption should enhance the description of dietary and other lifestyle interventions, thus allowing more objective comparisons between studies.

## Conclusions

In conclusion, this systematic review showed that RCTs of dietary interventions targeting people of retirement age used a range of BCTs, some of which were associated with greater intervention effectiveness. Dietary interventions in the retirement transition are likely to yield long-term health benefits and certain features of interventions, such as a greater number of contacts and inclusion of specific BCTs, notably ‘barrier identification/problem solving’, ‘plan social support/social change’, ‘goal setting (outcome)’ and ‘use of follow-up prompts’, appear to enhance the effectiveness of such interventions. These findings may help in developing more effective dietary interventions among individuals of retirement age.

## Additional file

Additional file 1: Table S1 Characteristics of RCTs and features of dietary interventions among adults of retirement age. **Figure S1.** Number of Behaviour change techniques used in the reviewed studies. **Figure S2.** F&V intakes in RCTs by the presence or absence of the “use of follow-up prompts” BCT. **Figure S3.** F&V intakes in RCTs by the presence or absence of the “goal setting (outcome)” BCT. **Figure S4.** F&V intakes in RCTs by the presence or absence of the “provide feedback on performance” BCT. **Figure S5.** F&V intakes in RCTs by the presence or absence of the “provide information on consequences of behaviour in general” BCT. **Figure S6.** F&V intakes in RCTs by the presence or absence of the “provide instruction on how to perform the behaviour” BCT. **Figure S7.** F&V intakes in RCTs by the presence or absence of the “model/demonstrate the behaviour” BCT. **Figure S8.** F&V intakes in RCTs by the presence or absence of the “goal setting (behaviour)” BCT. **Figure S9.** F&V intakes in RCTs by the presence or absence of the “Prompt review of behavioural goals” BCT. **Figure S10.** F&V intakes in RCTs by the presence or absence of the “Provide information on consequences of behaviour to the individual” BCT. **Figure S11.** F&V intakes in RCTs by the presence or absence of the “motivational interviewing” BCT.
