# Supplementary material for: Association of behaviour change techniques with effectiveness of dietary interventions among adults of retirement age: a systematic review and meta-analysis of randomised controlled trials
Source: BMC Med. 2014 Oct 7;12:177. doi: 10.1186/s12916-014-0177-3 (PMC4198739; doi:10.1186/s12916-014-0177-3)
Supplement: Additional file 1: Table S1 — Characteristics of RCTs and features of dietary interventions among adults of retirement age. Figure S1. Number of Behaviour change techniques used in the reviewed studies. Figure S2. F&V intakes in RCTs by the presence or absence of the “use of follow-up prompts” BCT. Figure S3. F&V intakes in RCTs by the presence or absence of the “goal setting (outcome)” BCT. Figure S4. F&V intakes in RCTs by the presence or absence of the “provide feedback on performance” BCT. Figure S5. F&V intakes in RCTs by the presence or absence of the “provide information on consequences of behaviour in general” BCT. Figure S6. F&V intakes in RCTs by the presence or absence of the “provide instruction on how to perform the behaviour” BCT. Figure S7. F&V intakes in RCTs by the presence or absence of the “model/demonstrate the behaviour” BCT. Figure S8. F&V intakes in RCTs by the presence or absence of the “goal setting (behaviour)” BCT. Figure S9. F&V intakes in RCTs by the presence or absence of the “Prompt review of behavioural goals” BCT. Figure S10. F&V intakes in RCTs by the presence or absence of the “Provide information on consequences of behaviour to the individual” BCT. Figure S11. F&V intakes in RCTs by the presence or absence of the “motivational interviewing” BCT. [file 12916_2014_177_MOESM1_ESM.doc]

**Additional file 1 (Supplementary material)**

**Table S1. Characteristics of RCTs and features of dietary interventions among adults of retirement age.**

| **Study** | **Age (years)**  **Mean±SD Gender**  **(% female)** | **Participants health status** | **Behaviour change techniques** CALO-RE taxonomy item | **Intervention** | **Participants Retention Rate (%)** | **Provider** | **Format** | **Setting** | **Intensity** | **Duration** |
| --- | --- | --- | --- | --- | --- | --- | --- | --- | --- | --- |
| Bemelmans *et al*. 2000 | 55±10  F/M (63) | High CVD risk | 1, 2, 5, 21, 22, 23, 27, 29 | Mediterranean diet | 80.5 | Dietitian for intensive group approach vs GP for usual care | Face to face –  group sessions by Dietitian vs Usual care in the form of a leaflet | Unclear | 3 group sessions of 2 hours each | Baseline march 2008  Follow-up at 16 and 52 weeks.  Plans for 105, 146 and 208 week follow-up |
| Bowen *et al*. 2009 | 54±16  F/M (85) | Healthy | 2, 5, 6, 21, 28, 37 | Intensive group education: three meetings of 2 hours each plus four booklets with core information about the education programme | 87.1 | Healthy eating coordinators | Mailed material  Social activities  Healthy eating sessions | Church/ community | Control group received standard educational materials.  Intervention group received culturally targeted self-help nutrition and PA materials, and 4 telephone counselling calls based on motivational interviewing delivered over the course of I year | 12 months |
| Campbell *et al*. 1999 | 54  F/M (73) | Low risk | 2, 5, 8, 19, 21, 22, 29 | Multilevel package to lower fat and increase fruit and vegetable consumption. | 100 | Trained interviewers  Mailed intervention  Pastors (sermons) | Posted printed material plus reinforcing messages from pastors | Home and Church | Monthly printed material; sermons at church reinforcing messages | 20 months |
| Coates *et al*. 1999 | 60  F(100) | Low risk | 1, 2, 5, 22 | Low-fat |  | Nutritionist | Face to face –  group sessions with Nutritionist | Clinical setting | Group sessions weekly for 6 weeks, biweekly for 6 weeks, monthly for 9 months, then quarterly. | 18 months |
| Estruch *et al*. 2013 | 67±6  F/M (53) | >3 CVD factors | 1, 2, 5, 7, 10, 19, 21, 25, 37 | Increasing fruit and vegetable intake. | 100 | Dietitian | Individual interviews  group sessions with written shopping lists meal plans etc | Clinical centres | Quarterly interviews  Quarterly group sessions  1 x follow up medical assessment | 12 months |
| Howard *et al* 2006 | 62±7  F (100) | Healthy | 1, 5, 6, 8, 16, 17, 19, 21, 26, 27, 28, 29, 33, 35, 36 | Multicomponent: Tailored bulletins, educational sessions, F&V availability at church, etc | 77.3 | Trained Nutritionists | Face to face group sessions | Clinical centres | 18 group sessions in year 1. Quarterly maintenance sessions thereafter (5 yrs) | 12 months |
| Iso *et al*. 2002 | 55±6  F/M (83) | Participants with hypercholesterolemia | 1, 5, 6, 7, 21, 27 | Mediterranean diet | 95 | Lectures by physicians, practical sessions by public health nurses & a nutritionist, telephone calls by publish health nurses | Face to face and telephone. Textbooks mailed to people who did not attend the classes | Education classes were held at a public health centre and two local community centres to which participants had easy access. | 7 education classes in first 6-months (1/2 h lecture plus practical nutrition sessions).  3 telephone calls & 1 education class in months 7-12. | 12 months |
| Kanaya *et al*. 2012 | 56±16  F/M (73) | Subjects at risk of DM | 5, 6, 27, 37 | Groups sessions + access to dietitian | 89 | Trained health department counsellors | Telephone-based counselling, 2 in-person sessions and 5 optional group workshops. | Community | The program consisted of 19 possible “contacts” for a total of 15 possible hours: 1 introductory session, which included a program binder; 1 in-person planning session; 12 telephone counselling calls (10 in active phase, 2 in maintenance phase); and 5 group workshops. | 12 months |
| Lanza *et al*. 2001 | 62±10  F/M (35) | Participants diagnosed with polyps in the previous 6 months | 1, 5, 6, 8, 13, 16, 19, 21, 22, 27, 29, | A dietary pattern lower in fat and higher in vegetables, fruit, and grains | 90.4 | Nutritionist | Face to face – individual & group  vs  general dietary guidelines from the National Dairy Council | Clinical centres | They had more than 50 hours of individual and group counselling sessions over 4 yrs | 48 months |
| Lapointe  *et al*. 2010 | 57±5  F (100) | Postmenopausal women | 1, 6, 21, 22 | Eighteen group-sessions/yr | 91.1 | Dietitian | Face to face – group and individual sessions | Unclear | 3 group sessions and ten individual sessions with a registered dietitian in 6 months | 6 months |
| Little *et al*. 2004 | 55±10  F/M (44) | High BP on a single occasion | 1, 5, 20, 27 |  | 90.2 | Nurse | Face to face | GP practices | Three face to face sessions; at baseline, 4 weeks and 6 months | 6 months |
| Marcus *et al*. 2001 | 54  F/M (80) | Callers to the NCI cancer information service | 1, 2, 5, 21, 23, | Reduce dietary intake of saturated fat and cholesterol, to increase intake of beans and vegetables, and to increase physical activity. | 90.5 | Information specialists at CIS centre | Telephone interview at baseline, 4 weeks, 4 months & 12 months. | Telephone interviews  (when the patient called the Cancer Information Service) | Baseline interview, then follow up at 4 weeks, 4 months & 12 months. Each phone call between 5-7 mins long | 12 months |
| Merril *et al*. 1999 | 57±9  F/M (35) | Healthy | 1, 5, 9, 21, 22 | A 1-year education program (8 classes/yr plus two telephone calls) | 100 | Dietitians, medical professionals e.g. nurses, Coronary Health Improvement Project (CHIP) staff | Face to face  scheduled shopping tours & cooking demos  Textbooks and workbooks | Clinical centre | Participants met for 4 weeks—four times each week for 2 hours on the CHIP study. Dietitians & medical professionals spoke weekly. Participants had access to scheduled shopping tours & cooking demos by the dietitian | 1 months |
| Panunzio *et al*. 2011 | 55±1  F/M (43) | Healthy | 1, 5, 6, 8, 10, 21, 22, 26, 29 | Education and skills training to modify diet and physical activity through primarily telephone-based counselling (12 calls) with 2 in-person sessions and 5 optional group workshops. |  | Nutritionist  Psychologists | Face to face – group and individual sessions | Unclear | 2-phases: Intensive phase of 15 weeks and Consolidation phase of 10 weeks.  Intensive phase: 5 weeks involving 10 (1-hr each) modules of a training course on lifestyle-related themes. 5 weeks of experiential “learn by doing” phase. And 5 weeks of 2 (1 hour each) modules per week.  Consolidation phase of 10 weeks consisting of 4 meetings (1 approximately every 17 days) directed to reinforce the changes achieved. | 25 weeks |
| Stevens *et al*. 2003 | 54±7  F (100) | Low risk | 5, 7, 8, 10, 19, 21, 37, | Increase fruits and vegetables and reduce fat. | 89 | Health counsellors | 20 min interactive touch-screen computer based | Clinical centre | Two 45-min counselling sessions (One of these including a 20-min interactive touch-screen computer based)  Two brief –telephone calls | 6-9 weeks |
| Takahashi *et al* 2006 | 56  F/M (67) | Healthy | 2, 5, 6, 19, 21, 27, | Intensive nutrition education and counselling program | 88 | Dietitian | Face to face counselling  Posted newsletters  1 x group lecture | Unclear | 2 x dietary counselling sessions (5 months o apart)  2 x newsletters  1 x group lecture (halfway) | 10 months |
| Tilley *et al* 1999 | 57±12  F/M (3) | High risk CRC | 1, 5, 19, 25 | Dietary intervention promoting high intakes of fruits and vegetables. | 91.4 | Unclear | Face to face groups classes | Worksite | (Year 1)  5 nutrition classes  Mailed self-help material  (Year 2)  Worksite posters, personalised feedback on FFQ | 24 months |
| Van keulen *et al*. 2011 | 57±7  F/M (45) | 52% hypertensive subjects | 1, 2, 5, 19, 37, |  | 93.9 | Trained interviewer counsellors | Tailored print communication;  Telephone motivational interviewing;  Combination of these | Mail and/or telephone delivered intervention | Tailored print communication: 4 tailored letters (#1 and #3  focused on PA and #2 and #4 on fruit and vegetable consumption)  Telephone motivational interviewing: 4 telephone  calls based on motivational interviewing. Participants chose  the order of the conversation topics in #1 and #3; if PA was  preferred in #1, fruit and vegetable consumption was discussed in #2, and vice versa.  Combination of these: 2 tailored print letters and 2 telephone  motivational interviews; #1 (letter) and #2 (interview) addressed PA, #3 (letter) and #4 (interview) focused on fruit and vegetable consumption. | 73 weeks |
| Walker *et al* 2009 | 58±5  F (100) | Low risk | 1, 2, 5, 6, 8, 19, 22 27, 23, 28, |  |  | Nurse involved in supervised self-report questionnaires | Mail | Community/home | 18 newsletters in 12 months (every 2 wks during first 6 months; every 4 wks months 7-12)  Feedback on diet? PA at 1, 3, 6 and 9 months  Motivational devices: Food pyramid magnets for fridges and pedometers | 12 months |
| Werkman *et al*. 2010 | 60±3  M (0) | Healthy | 1, 2, 4, 6, 16, 19, 21 |  |  | Unclear | Internet  And printed material | unclear | Five programme modules were provided to participants  of the intervention group during the 1-yr intervention. Participants could freely choose to make use of the modules or not. In addition, the intervention group received newsletters every 2-3 months that contained study information, information about diet and physical activity and encouragements to use the modules. | 12 months |
| Wolf *et al*. 2009 | 55±6  (study included few women, but published results include only men) | Low risk | 1, 2, 5, 6, 27, |  |  | Health educators | Two telephone calls  Mailed brochure | Community/Home | Two telephone calls within a month. One educational call; one follow-up call. | 1 months |
| Wright *et al*. 2011 | 56±7  M (0) | Participants having one or more CVD risk factors | 1, 4, 5, 8, 10, 13, 19, |  |  | Dietitian | 1) tailored, iterative, printed dietary feedback  2) small group nutrition education sessions dietitian-led | University seminar rooms | 1) tailored, iterative, printed dietary feedback (TF) with three instalments mail-delivered over a 3-month period that were re-tailored to most recent assessment of dietary intake, intention to change and assessment of self-adequacy of dietary intake.  2) small group nutrition education sessions (GE): consisting of two 90-minute dietitian-led small group sessions l | 3 months |

**Behaviour change techniques CALO-RE taxonomy item**: (1) Provide information on consequences of behaviour in general, (2) Provide information on consequences of behaviour to the individual, (4) Provide normative information about others’ behaviour, (5) Goal setting behaviour, (6) Goal setting outcome, (7) Action planning, (8) Barrier identification/problem solving, (9) Set graded tasks, (10) Prompt review of behavioural goals, (13) Provide rewards contingent on successful behaviour, (16) Prompt self-monitoring of behaviour, (17) Prompt self-monitoring of behavioural outcome, (19) Provide feedback on performance, (20) Provide information on where and when to perform the behaviour, (21) Provide instruction on how to perform the behaviour, (22) Model/Demonstrate the behaviour, (23) Teach to use prompts/cues, (24) Environmental restructuring, (25) Agree behavioural contract, (26) Prompt practice, (27) Use of follow-up prompts, (28) Facilitate social comparison, (29) Plan social support/social change, (33) Prompt self talk, (35) Relapse prevention/coping planning, (36) Stress management/emotional control training, (37) Motivational interviewing, (38) Time management.

**Figure S1**. **Number of Behaviour change techniques used in the reviewed studies**

Median =6

**Figure S2.** **F&V intakes in RCTs by the presence or absence of the “use of follow-up prompts” BCT**


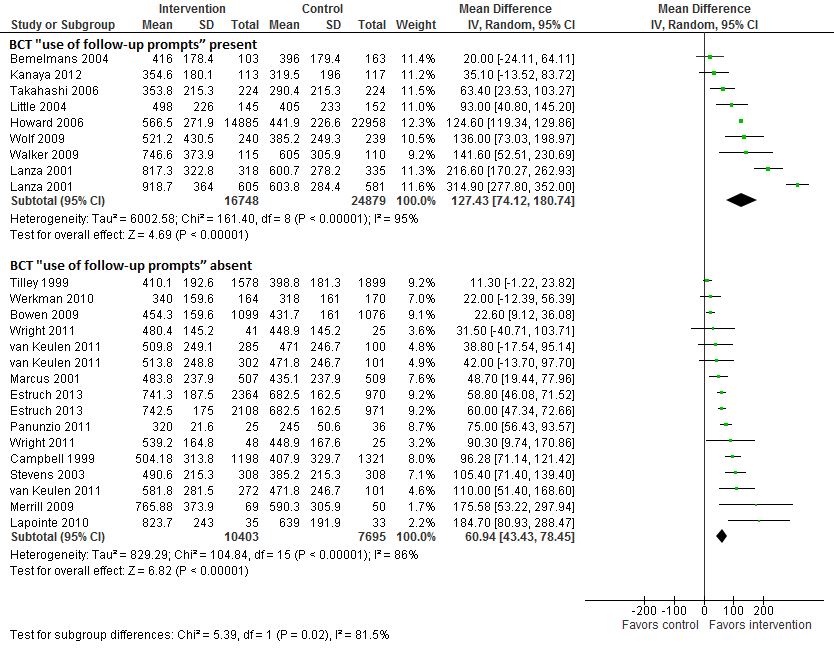


**Figure S3.** **F&V intakes in RCTs by the presence or absence of the “goal setting (outcome)” BCT**


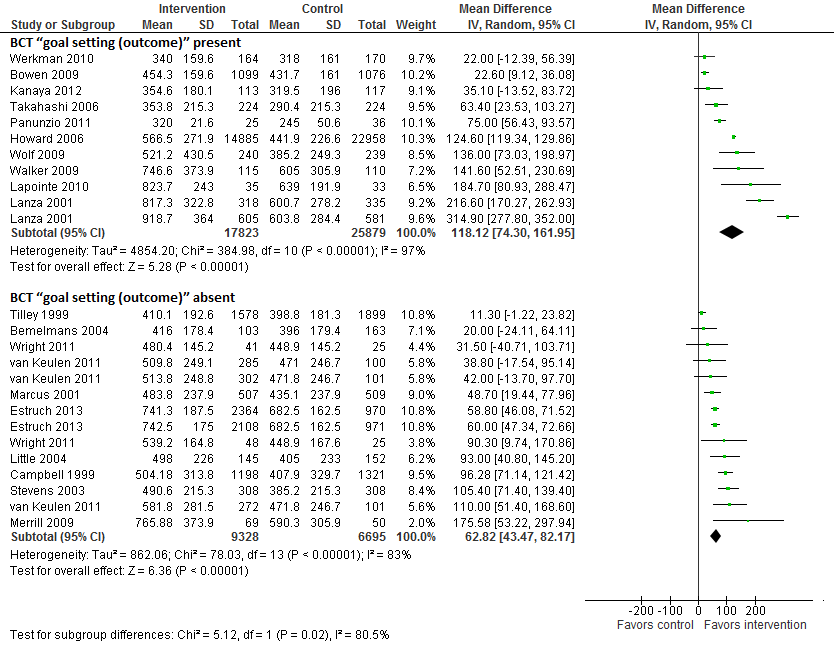


**Figure S4.** **F&V intakes in RCTs by the presence or absence of the “provide feedback on performance” BCT**


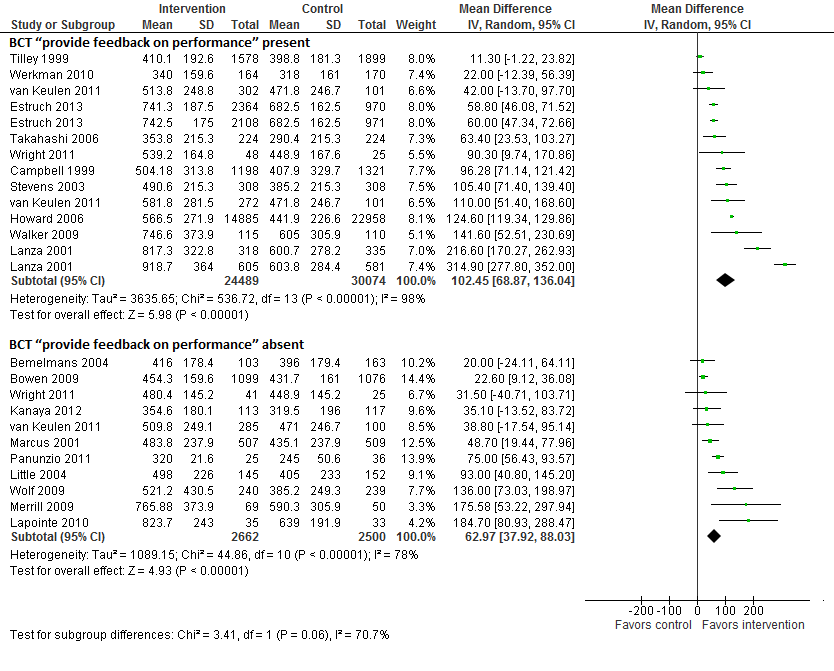


**Figure S5.** **F&V intakes in RCTs by the presence or absence of the “provide information on consequences of behaviour in general” BCT**


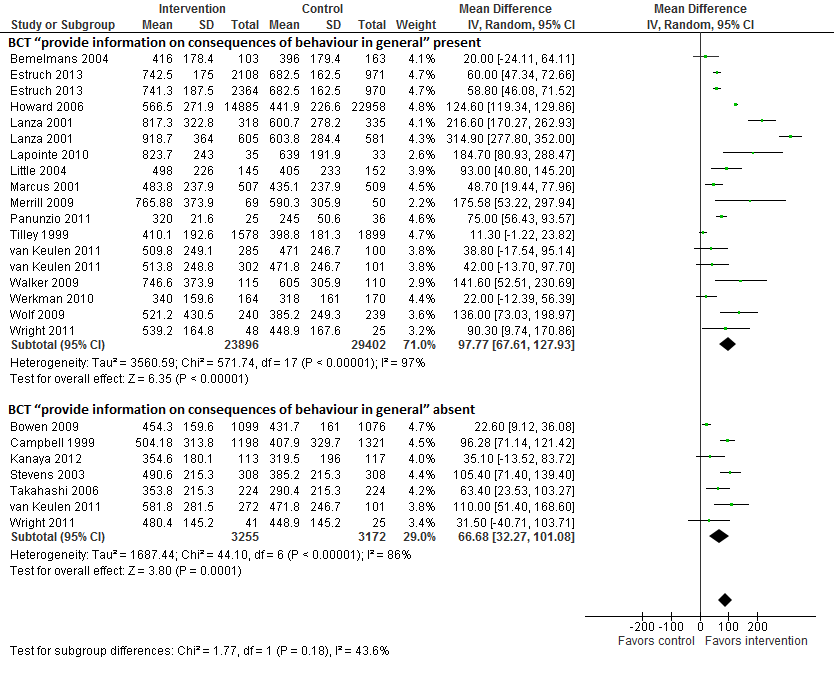


**Figure S6.** **F&V intakes in RCTs by the presence or absence of the “provide instruction on how to perform the behaviour” BCT**


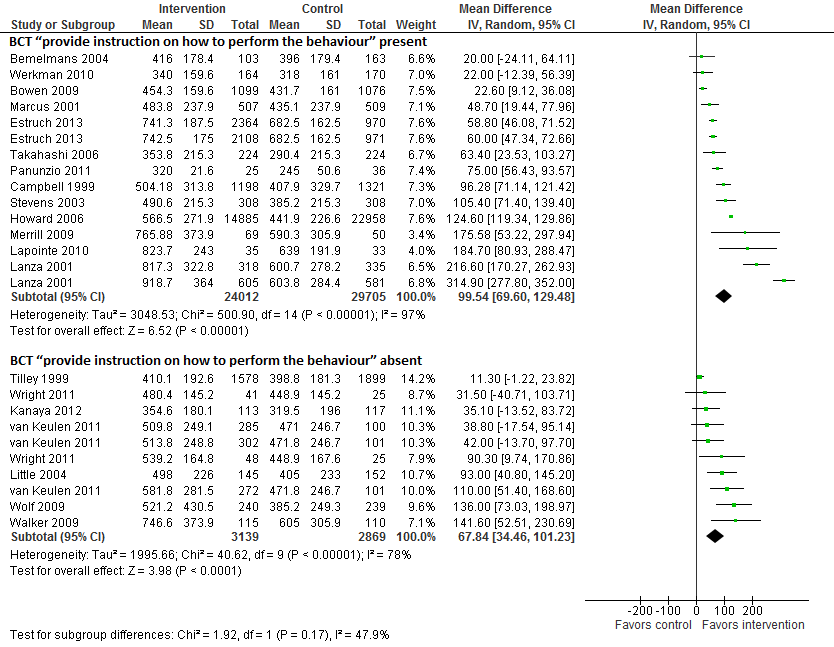


**Figure S7.** **F&V intakes in RCTs by the presence or absence of the “model/demonstrate the behaviour” BCT**


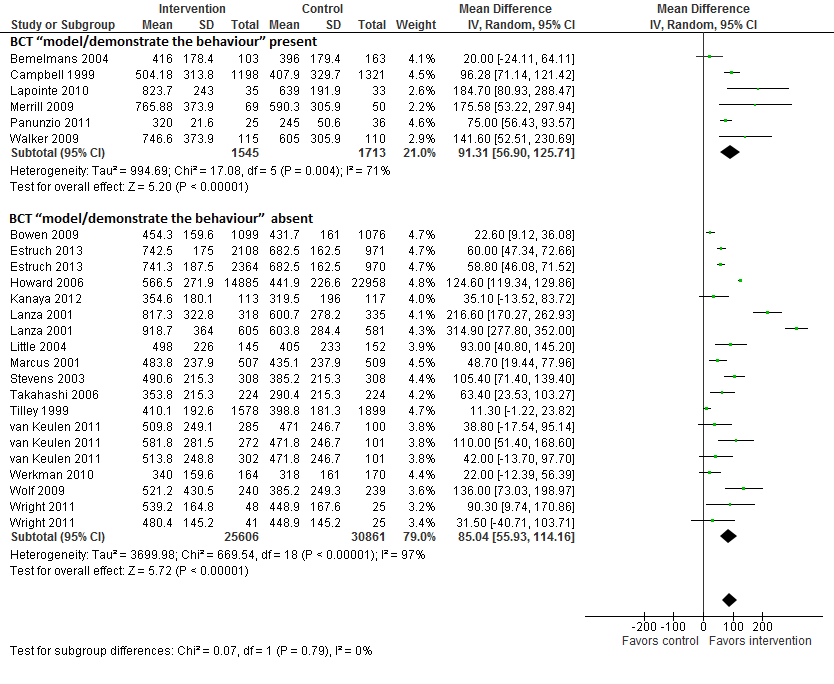


**Figure S8.** **F&V intakes in RCTs by the presence or absence of the “goal setting (behaviour)” BCT**


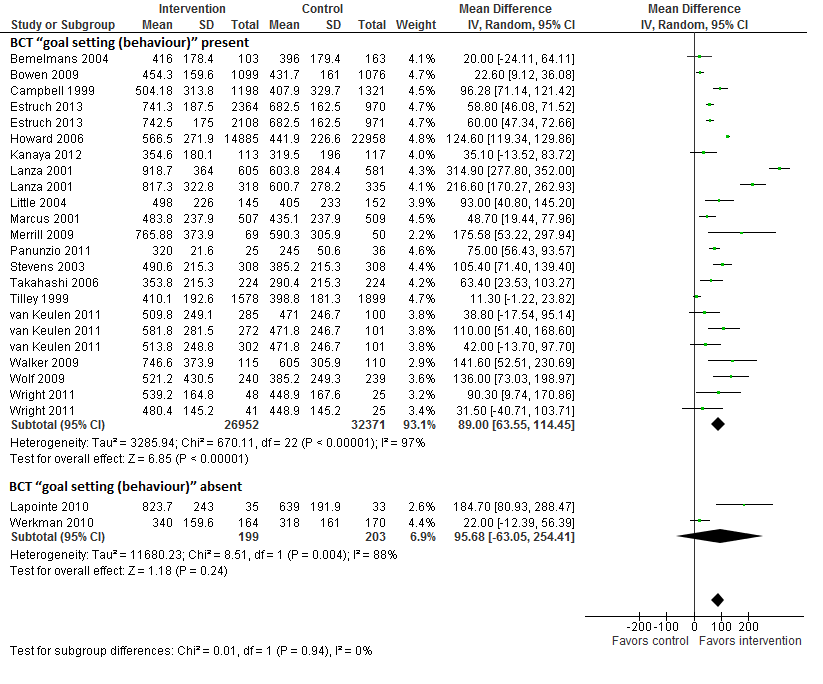


**Figure S9.** **F&V intakes in RCTs by the presence or absence of the “Prompt review of behavioural goals” BCT**


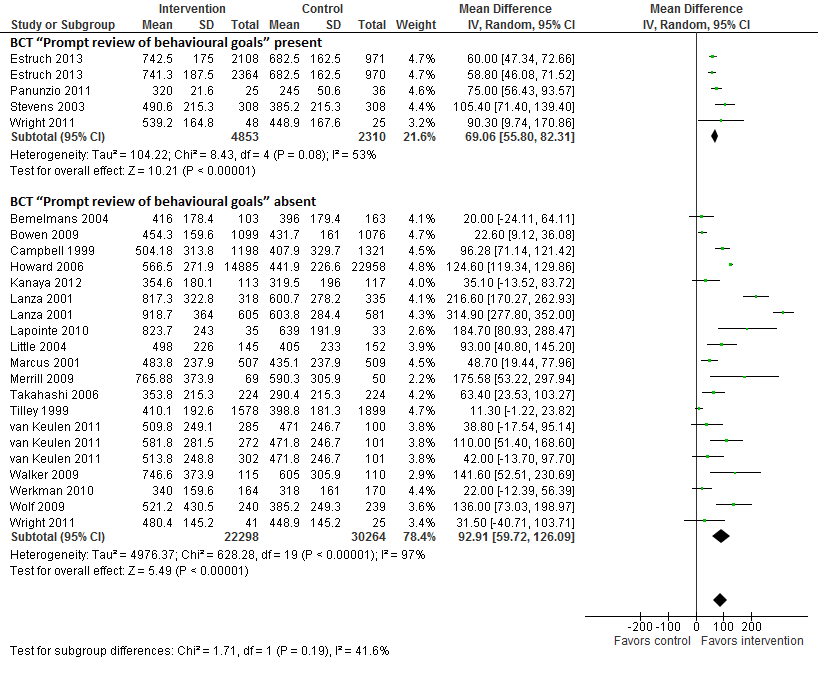


**Figure S10.** **F&V intakes in RCTs by the presence or absence of the “Provide information on consequences of behaviour to the individual” BCT**


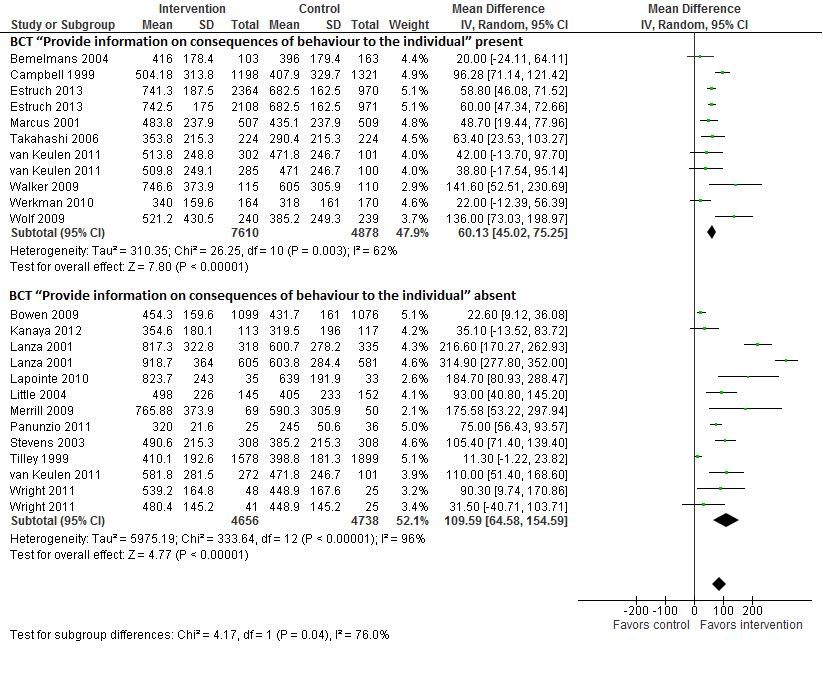


**Figure S11.** **F&V intakes in RCTs by the presence or absence of the “motivational interviewing” BCT**


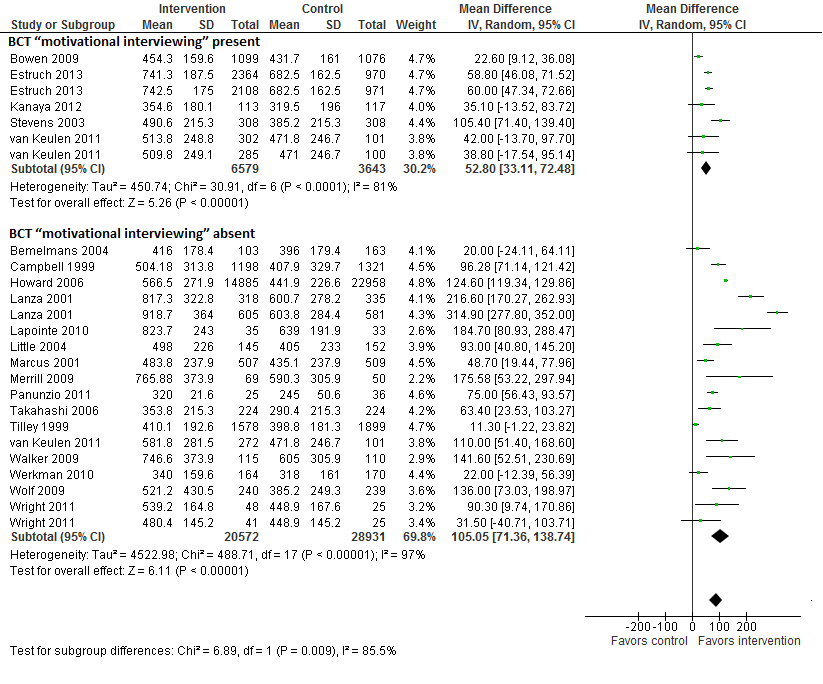


1. Bemelmans WJ, Broer J, de Vries JH, Hulshof KF, May JF, Meyboom-De Jong B: **Impact of Mediterranean diet education versus posted leaflet on dietary habits and serum cholesterol in a high risk population for cardiovascular disease**. *Public health nutrition* 2000, **3**(3):273-283.

2. Bowen DJ, Beresford SA, Christensen CL, Kuniyuki AA, McLerran D, Feng Z, Hart A, Jr., Tinker L, Campbell M, Satia J: **Effects of a multilevel dietary intervention in religious organizations**. *American journal of health promotion : AJHP* 2009, **24**(1):15-22.

3. Campbell MK, Demark-Wahnefried W, Symons M, Kalsbeek WD, Dodds J, Cowan A, Jackson B, Motsinger B, Hoben K, Lashley J *et al*: **Fruit and vegetable consumption and prevention of cancer: the Black Churches United for Better Health project**. *American journal of public health* 1999, **89**(9):1390-1396.

4. Coates RJ, Bowen DJ, Kristal AR, Feng Z, Oberman A, Hall WD, George V, Lewis CE, Kestin M, Davis M *et al*: **The Women's Health Trial Feasibility Study in Minority Populations: Changes in Dietary Intakes**. *American journal of epidemiology* 1999, **149**(12):1104-1112.

5. Estruch R, Ros E, Salas-Salvado J, Covas MI, Corella D, Aros F, Gomez-Gracia E, Ruiz-Gutierrez V, Fiol M, Lapetra J *et al*: **Primary prevention of cardiovascular disease with a Mediterranean diet**. *The New England journal of medicine* 2013, **368**(14):1279-1290.

6. Howard BV, Curb JD, Eaton CB, Kooperberg C, Ockene J, Kostis JB, Pettinger M, Rajkovic A, Robinson JG, Rossouw J *et al*: **Low-fat dietary pattern and lipoprotein risk factors: the Women's Health Initiative Dietary Modification Trial**. *The American journal of clinical nutrition* 2010, **91**(4):860-874.

7. Iso H, Imano H, Nakagawa Y, Kiyama M, Kitamura A, Sato S, Naito Y, Shimamoto T, Iida M: **One-year community-based education program for hypercholesterolemia in middle-aged Japanese: a long-term outcome at 8-year follow-up**. *Atherosclerosis* 2002, **164**(1):195-202.

8. Kanaya AM, Santoyo-Olsson J, Gregorich S, Grossman M, Moore T, Stewart AL: **The Live Well, Be Well Study: A Community-Based, Translational Lifestyle Program to Lower Diabetes Risk Factors in Ethnic Minority and Lower–Socioeconomic Status Adults**. *American journal of public health* 2012, **102**(8):1551-1558.

9. Lanza E, Schatzkin A, Daston C, Corle D, Freedman L, Ballard-Barbash R, Caan B, Lance P, Marshall J, Iber F *et al*: **Implementation of a 4-y, high-fiber, high-fruit-and-vegetable, low-fat dietary intervention: results of dietary changes in the Polyp Prevention Trial**. *The American journal of clinical nutrition* 2001, **74**(3):387-401.

10. Lapointe A, Weisnagel SJ, Provencher V, Begin C, Dufour-Bouchard AA, Trudeau C, Lemieux S: **Using restrictive messages to limit high-fat foods or nonrestrictive messages to increase fruit and vegetable intake: what works better for postmenopausal women?** *European journal of clinical nutrition* 2010, **64**(2):194-202.

11. Little P, Kelly J, Barnett J, Dorward M, Margetts B, Warm D: **Randomised controlled factorial trial of dietary advice for patients with a single high blood pressure reading in primary care**. *BMJ* 2004, **328**(7447):1054.

12. Marcus AC, Heimendinger J, Wolfe P, Fairclough D, Rimer BK, Morra M, Warnecke R, Himes JH, Darrow SL, Davis SW *et al*: **A randomized trial of a brief intervention to increase fruit and vegetable intake: a replication study among callers to the CIS**. *Preventive medicine* 2001, **33**(3):204-216.

13. Merrill RM, Aldana SG: **Consequences of a plant-based diet with low dairy consumption on intake of bone-relevant nutrients**. *J Womens Health (Larchmt)* 2009, **18**(5):691-698.

14. Panunzio MF, Caporizzi R, Antoniciello A, Cela EP, Ferguson LR, D'Ambrosio P: **Randomized, controlled nutrition education trial promotes a Mediterranean diet and improves anthropometric, dietary, and metabolic parameters in adults**. *Ann Ig* 2011, **23**(1):13-25.

15. Stevens VJ, Glasgow RE, Toobert DJ, Karanja N, Smith KS: **One-year results from a brief, computer-assisted intervention to decrease consumption of fat and increase consumption of fruits and vegetables**. *Preventive medicine* 2003, **36**(5):594-600.

16. Takahashi Y, Sasaki S, Okubo S, Hayashi M, Tsugane S: **Blood pressure change in a free-living population-based dietary modification study in Japan**. *Journal of hypertension* 2006, **24**(3):451-458.

17. Tilley BC, Glanz K, Kristal AR, Hirst K, Li S, Vernon SW, Myers R: **Nutrition intervention for high-risk auto workers: results of the Next Step Trial**. *Preventive medicine* 1999, **28**(3):284-292.

18. van Keulen H, Mesters I, Ausems M, Breukelen G, Campbell M, Resnicow K, Brug J, Vries H: **Tailored Print Communication and Telephone Motivational Interviewing Are Equally Successful in Improving Multiple Lifestyle Behaviors in a Randomized Controlled Trial**. *ann behav med* 2011, **41**(1):104-118.

19. Walker SN, Pullen CH, Boeckner L, Hageman PA, Hertzog M, Oberdorfer MK, Rutledge MJ: **Clinical trial of tailored activity and eating newsletters with older rural women**. *Nursing research* 2009, **58**(2):74-85.

20. Werkman A, Hulshof PJ, Stafleu A, Kremers SP, Kok FJ, Schouten EG, Schuit AJ: **Effect of an individually tailored one-year energy balance programme on body weight, body composition and lifestyle in recent retirees: a cluster randomised controlled trial**. *BMC public health* 2010, **10**:110.

21. Wolf RL, Lepore SJ, Vandergrift JL, Basch CE, Yaroch AL: **Tailored telephone education to promote awareness and adoption of fruit and vegetable recommendations among urban and mostly immigrant black men: a randomized controlled trial**. *Preventive medicine* 2009, **48**(1):32-38.

22. Wright J, Sherriff J, Dhaliwal S, Mamo J: **Tailored, iterative, printed dietary feedback is as effective as group education in improving dietary behaviours: results from a randomised control trial in middle-aged adults with cardiovascular risk factors**. *International Journal of Behavioral Nutrition and Physical Activity* 2011, **8**(1):43.
